# Initial Presentation Sites as Predictors of Herpes Zoster Complications: A Nationwide Cohort Study

**DOI:** 10.1371/journal.pone.0164019

**Published:** 2016-10-06

**Authors:** Wen-Yi Wang, Sing-Huh Liu, Meng-Yin Lin, Che-Chen Lin, I-Jong Wang

**Affiliations:** 1 Department of Ophthalmology, National Taiwan University Hospital, Taipei, Taiwan; 2 Department of Ophthalmology, Shuang Ho Hospital, Taipei Medical School of Medicine, Taipei Medical University, Taipei County, Taiwan; 3 Institute of Clinical Medicine, School of Medicine, National Taiwan University, Taipei, Taiwan; 4 Management Office for Health Data, China Medical University Hospital, Taichung, Taiwan; 5 School of Medicine, China Medical University, Taichung, Taiwan; 6 Institute of Clinical Medicine, School of Medicine, China Medical University, Taichung, Taiwan; University of Illinois at Chicago, UNITED STATES

## Abstract

Herpes zoster (HZ) is associated with complications such as postherpetic neuralgia (PHN) and HZ ophthalmicus (HZO). However, few studies have focused on identifying patients having a high risk of PHN and HZO according to the initial presentation sites. The current study investigated these factors in a nationwide population-based cohort derived from Taiwan’s Longitudinal Health Insurance Database. The results indicate that the initial presentation sites can predict the complication site of HZ. In this study, elderly patients were found to be more susceptible to HZ and were the first to present with neurological signs (HZN). Furthermore, compared with patients with HZO and other signs (HZT), those with HZN had a higher comorbidity risk. Patients with HZN showed a significantly higher visceral complication risk than did those with HZO (adjusted hazard ratio [HR] = 1.47, 95% confidence interval [CI] = 1.27–1.71). In addition, patients with HZT showed lower risks of ocular and neurological complications than did those with HZN after stratification by age and sex (adjusted HR = 0.46, 95% CI = 0.31–0.68 and HR = 0.73, 95% CI = 0.59–0.91, respectively).

## Introduction

Reactivation of the latent varicella—zoster virus (VZV) in the cranial nerve or dorsal root ganglia leads to herpes zoster (HZ), also known as shingles [1]. Typically, HZ causes unilateral cutaneous pain and blistering rashes that are limited to one dermatome [1]. The estimated incidence rate of HZ is 30%. However, approximately 50% of unvaccinated people aged ≤85 years develop HZ [2–4]. The global incidence rate of HZ is 3–5/1000 person-years, sharply increasing after the age of 50 years [3]. The global incidence rate is estimated to be approximately 6–8 and 10–12/1000 person-years in people aged 60 and ≥80 years, respectively [3, 5].

HZ is common and increasingly prevalent; it causes debilitating pain, neuropathy, and inflammatory complications, leading to high health care expenditure and productivity loss [2, 4, 6–8]. Moreover, accumulating evidence has indicated that HZ may increase the risk of other severe conditions, particularly stroke [9, 10]. HZ is associated with complications, mainly postherpetic neuralgia (PHN) and HZ ophthalmicus (HZO) [3, 11]. Approximately 10%–18% of patients with HZ develop PHN, which is characterized by persistent pain after the resolution of the rash [12]. However, >30% of patients with PHN experience pain lasting for >1 year [3]. The prevalence of HZO is 10%–25% among patients with HZ, arising because of reactivation of the latent virus in the trigeminal ganglia involved in the ophthalmic division of the trigeminal nerve [9]. Patients with HZO require medical care for an average of 10 months, and 6% of these patients develop vision loss [13].

HZ occurs in patients with poor cell-mediated immunity [14], which diminishes with age and immune deficiency or suppression [12, 15]. Patients aged >50 years who take immunosuppressive drugs, have HIV/AIDS, or have undergone bone marrow or organ transplants have a high risk of HZ [5, 16, 17]. Other risk factors include cancer, chronic steroid therapy, psychological stress, sex, ethnicity, primary infection during infancy or in utero, and trauma [15, 18–20]. Moreover, HZ complications are intolerable and result in patient disability. Therefore, elucidating the risk factors for extremely painful and vision-threatening complications is imperative for identifying patients having a high risk of HZ and its complications. Many studies have indicated that greater acute pain and rash severities and prodromal pain are predictors of PHN [21–27]. However, few population-based studies have focused on HZO [11]. Moreover, according to our review of relevant literature, no studies have yet identified patients at high risks of PHN and HZO according to the initial presentation sites.

Therefore, this study investigated the association between the initial presentation and complication sites of HZ in a nationwide population-based cohort in Taiwan. Moreover, different complications in the cohorts were evaluated after stratification by age and sex.

## Methods

### Data source

The study population was derived from the Longitudinal Health Insurance Database (LHID). The LHID contains the data of one million randomly selected enrollees of the National Health Insurance (NHI) program, a single-payer, mandatory health insurance program covering the 23 million residents of Taiwan, for the period 1996–2000. According to a report published by the Taiwan government, age and sex distributions in the LHID and NHI program do not differ significantly. The LHID comprises the claims data of the NHI program, including the registry of beneficiaries; inpatient and outpatient files, in which the disease history is recorded using International Classification of Diseases, Ninth Revision, Clinical Modification [ICD-9-CM] codes; and data on other medical services. The LHID was released for research after encrypting the original identification numbers of the insurance beneficiaries to protect patient privacy. This study was approved by the Ethics Review Board of China Medical University (CMUH104-REC2-115).

### Study population

This retrospective population-based cohort study elucidated the association between the risk of HZ complications and types of HZ. The study cohort comprised patients who had newly developed HZ (ICD-9-CM 053) between January 1, 2000 and December 31, 2009. Patients who received previous HZ coding within 180 days before the index date were excluded, and those with missing information on sex or age were not enrolled. The patients were classified into the following cohorts. 1) HZO: patients with an initial ICD-9-CM codes of 053.20, 053.21, 053.22, or 053.29; 2) HZN: patients with initial ICD-9-CM codes of 053.11, 053.0, 053.71, and 053.19; and 3) HZT: patients with ICD-9-CM codes different from those for HZO and HZN. The index date in the HZ cohort was the date of the initial HZ diagnosis. All patients who experienced HZ complications before the index date were excluded from the study. The following HZ complications were studied: cutaneous (ICD-9-CM 680–686, 035, 728, 034, and 041); ocular (ICD-9-CM 379.00, 379.09, 373.00, 376.01, 376.00, 376.10, 365.60, 370.35, 364.10, 364.00, 362.89, 363.20, 360.12, 363.21, 370.00, 371.00, 370.50, 370.35, 370.60, and 371.43 or HZ coding changed to ICD-9-CM 053.2); neurological (ICD-9-CM 320–323, 342, 348, 351, 378, 438.2, 780.3, and V17.1 or HZ coding changed to ICD-9-CM 053.0 and 053.1); primary trigeminal neuralgia (PTN, with PTN drug use); and other visceral (ICD-9-CM 038, 040, 041, 070.5, 070.9, 730–733, 480–487, 466, 490, 422, 425, 510–519, 287, 790.7, 995.91, 995.92, 573, and 577.0 or HZ coding changed to ICD-9-CM 053.8, 053.79, and 053.71) outcomes. Each patient was followed until the occurrence of an HZ complication; withdrawal from the NHI program; or December 31, 2011.

The following comorbidities were considered confounders: coronary artery disease (ICD-9-CM 410–414), hypertension (ICD-9-CM 401–405), hyperlipidemia (ICD-9-CM 272), diabetes mellitus (DM, ICD-9-CM250), stroke (ICD-9-CM 430–438), atrial fibrillation (AF; ICD-9-CM 427.31), renal disease (ICD-9-CM 580–589), heart failure (ICD-9-CM 428), cancer (ICD-9-CM 140–208), chronic hepatitis (ICD-9-CM 571, 572.2, 572.3, 572.8, 573.1–573.3, 573.8, and 573.9), and systemic lupus erythematosus (SLE, ICD-9-CM 710).

### Statistical analysis

The mean age and corresponding standard deviation were calculated, and the age difference in the HZ cohorts was determined using an analysis of variance test. In addition, the number and percentage for sex and comorbidities were calculated, and the differences in their distribution in the cohorts were assessed using the chi-squared test. The incidence density of each HZ complication in each HZ cohort was calculated as the total number of complication events divided by the sum of the follow-up period (per 1000 person-years). Furthermore, the overall cumulative incidence of HZ complications in each HZ cohort was measured using the Kaplan—Meier method, and the curve differences were determined using the log-rank test. To estimate the risk of HZ complications in the HZ cohorts, the hazard ratios (HRs) and 95% confidence intervals (CIs) were measured using crude and adjusted Cox proportional hazards models. The risk of HZ complications in the HZO, HZN, and HZT cohorts was analyzed after stratification by age and sex. SAS version 9.4 (SAS Institute, Cary, NC, USA) was used for data management and integration and for all statistical analyses. The statistical significance was set at p < 0.05.

## Results

Table 1 lists the studied characteristics of the three cohorts. The HZO, HZN, and HZT cohorts comprised 660, 1388, and 22,495 patients, respectively. The mean patient ages in the HZO, HZN, and HZT cohorts were 57.3, 57.6, and 54.9 years, respectively. The sex ratio did not significantly differ among the three cohorts (p = 0.2783). In addition, the proportion of patients with DM, AF, heart failure, chronic hepatitis, and SLE did not significantly differ among the cohorts.

**Table 1:** Demographic status and comorbidities in the study cohorts.

Variable	HZO, N = 660 (%)	HZN, N = 1388 (%)	HZT, N = 22495 (%)	p-value
Age, years (SD)^†^	57.3 (17.5)	57.6 (16.5)	54.9 (16.7)	<0.0001
Sex				
Female	341 (51.7)	715 (51.5)	12013 (53.4)	0.2783
Male	319 (48.3)	673 (48.5)	10482 (46.6)	
Comorbidity				
CAD	188 (28.5)	430 (31.0)	5403 (24.0)	<0.0001
Hypertension	299 (45.3)	645 (46.5)	9013 (40.1)	<0.0001
Hyperlipidemia	190 (28.8)	484 (34.9)	6593 (29.3)	<0.0001
DM	107 (16.2)	221 (15.9)	3257 (14.5)	0.1666
Stroke	38 (5.8)	85 (6.1)	966 (4.3)	0.0014
AF	16 (2.4)	33 (2.4)	379 (1.7)	0.0641
Renal disease	104 (15.8)	248 (17.9)	2821 (12.5)	<0.0001
Heart failure	48 (7.3)	112 (8.1)	1237 (5.5)	<0.0001
Cancer	32 (4.8)	68 (4.9)	980 (4.4)	0.5383
Chronic hepatitis	212 (32.1)	452 (32.6)	6872 (30.5)	0.2085
SLE	1 (0.2)	6 (0.4)	89 (0.4)	0.5931

^†^ANOVA test.

Abbreviations: CAD: coronary artery disease (ICD-9-CM 410–414); DM: diabetes mellitus (ICD-9-CM 272); AF: atrial fibrillation (ICD-9-CM 427.31); SLE: systemic lupus erythematosus (ICD-9-CM 710), hypertension (ICD-9-CM 401–405), hyperlipidemia (ICD-9-CM 272), stroke (ICD-9-CM 430–438), renal disease (ICD-9-CM 580–589), heart failure (ICD-9-CM 428), cancer (ICD-9-CM 140–208), and chronic hepatitis (ICD-9-CM 571, 572.2, 572.3, 572.8, 573.1–573.3, 573.8, and 573.9)

**Fig 1:**
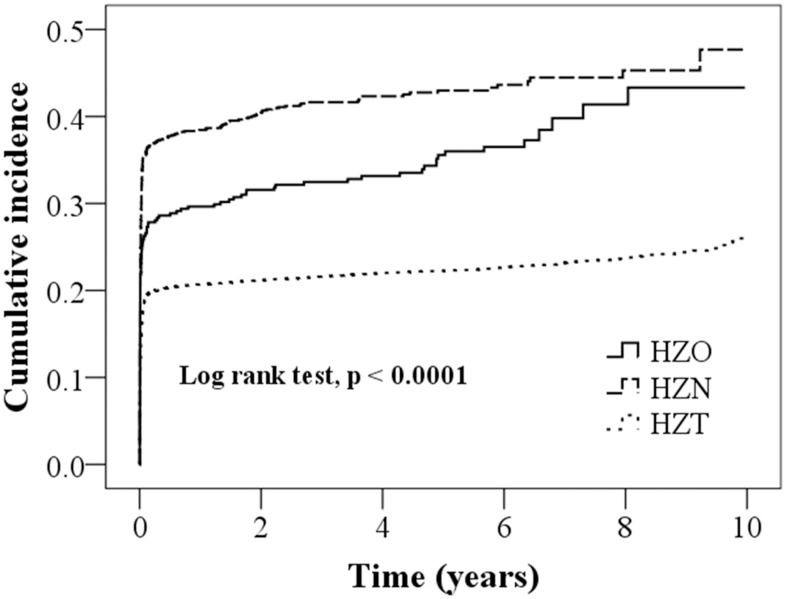
Overall cumulative risks of complications in the HZO, HZN, and HZT cohorts.

Table 2 lists the risk of HZ complications in all cohorts. Fig 1 shows a significant overall incidence of HZ complications in the HZ cohorts (log-rank test, p < 0.0001). The incidence of cutaneous complications in the HZO, HZN, and HZT cohorts was 7.77, 7.46, and 6.16/1000 person-years, respectively. Compared with the HRs in the HZO cohort, the HRs for cutaneous outcomes were 0.90 (95% CI = 0.58–1.40) and 0.84 (95% CI = 0.58–1.21) in the HZN and HZT cohorts, respectively. After adjustment for age, sex, and comorbidities, the HR for PTN was 1.20 (95% CI = 0.83–1.74) in the HZN cohort and 0.93 (95% CI = 0.68–1.29) in the HZT cohort compared with the HZO cohort. The HZT cohort showed a 0.46-fold lower risk of ocular complications relative to that of the HZN cohort (HR = 0.46, 95% CI = 0.31–0.68). The HZT cohort had a 0.73-fold lower risk of neurological complications than did the HZO cohort (HR = 0.73, 95% CI = 0.59–0.91). In addition, the HZN cohort was more significantly associated with an increased risk of other visceral complications than was the HZO cohort (HR = 1.47, 95% CI = 1.27–1.71).

**Table 2:** Adjusted hazard ratios (95% confidence interval) for herpes zoster complication risk. Adjusted HR: Adjusted for age, sex, CAD, hypertension, hyperlipidemia, DM, stroke, AF, renal disease, heart failure, cancer, chronic hepatitis, and SLE. Abbreviations: CAD: coronary artery disease; DM: diabetes mellitus; AF: atrial fibrillation; SLE: systemic lupus erythematosus; Rate: incidence, per 1000 person-years; PTN: primary trigeminal neuralgia.

	Type of HZ								
Type of outcome	HZO			HZN			HZT		
	Event	Rate	Adjusted HR (95% CI)	Event	Rate	Adjusted HR (95% CI)	Event	Rate	Adjusted HR (95% CI)
Cutaneous	30	7.77	ref	58	7.46	0.90(0.58–1.40)	805	6.16	0.84(0.58–1.21)
Ocular	-	-	-	29	3.65	ref	205	1.53	0.46(0.31–0.68)
Neurologic	82	22.9	ref	-	-	-	1885	15.1	0.73(0.59–0.91)
PTN	39	10.1	ref	98	12.6	1.20(0.83–1.74)	1163	8.90	0.93(0.68–1.29)
Other visceral	229	79.9	ref	651	143	1.47(1.27–1.71)	-	-	-

Tables 3–7 shows the risk of HZ complications in the HZ cohorts after stratification by age and sex. Table 3 shows the risk of cutaneous outcomes in the cohorts. The risk of cutaneous outcomes in the HZN and HZT cohorts was not significantly different compared with that in the HZO cohort after stratification by age and sex. Table 4 lists the ocular risk in the study cohorts stratified by age and sex. Compared with the HRs in the HZN cohort, those for ocular complications were 0.54 (95% CI = 0.30–0.99) for female patients and 0.40 (95% CI = 0.24–0.68) for male patients in the HZT cohort. Except for patients in the ≥65-year age group, those in all other age groups in the HZT cohort exhibited a significantly lower risk of ocular complications than did those in the <45-year (HR = 0.20, 95% CI = 0.09–0.43) and 45–64-year (HR = 0.37, 95% CI = 0.20–0.69) age groups in the HZN cohort. Table 5 presents the risk of neurological complications in the HZO and HZT cohorts stratified by age and sex. The risk of neurological complications was significantly lower in the HZT cohort than in the HZO cohort only for patients aged 45–64 years (HR = 0.55, 95% CI = 0.38–0.80) and male patients (HR = 0.62, 95% CI = 0.46–0.82). Table 6 presents the PTN outcomes for all HZ cohorts. Compared with the risk of PTN in the HZO cohort, that in the HZN and HZT cohorts stratified by age and sex was nonsignificant. Compared with the HRs for other visceral complications in the HZO cohort (Table 7), those in the <45, 45–64, and ≥65-year age groups of the HZN cohort were 1.68 (95% CI = 1.17–2.39), 1.55 (95% CI = 1.21–2.00), and 1.34 (95% CI = 1.07–1.68), respectively. Male and female patients in the HZN cohort showed 1.76-fold (95% CI = 1.41–2.18) and 1.24-fold (95% CI = 1.00–1.53) increased risks of other visceral complications, respectively, compared with those in the HZO cohort.

**Table 3:** Risk of herpes zoster complications (cutaneous) in types of herpes zoster stratified by age and sex. Adjusted HR: Adjusted for age, sex, CAD, hypertension, hyperlipidemia, DM, stroke, AF, renal disease, heart failure, cancer, chronic hepatitis, and SLE. Rate: incidence rate per 1000 person-years.

Variable	HZO			HZN			HZT		
	Event	Rate	Adjusted HR (95% CI)	Event	Rate	Adjusted HR (95% CI)	Event	Rate	Adjusted HR (95% CI)
Age group									
<45	2	1.84	ref	7	3.76	1.84(0.38–8.89)	131	3.38	1.70(0.42–6.87)
45–64	12	8.76	ref	18	5.26	0.58(0.28–1.20)	292	5.11	0.57(0.32–1.02)
≧65	16	11.4	ref	33	13.2	1.06(0.58–1.93)	382	11.0	0.94(0.57–1.56)
Sex									
Female	12	5.79	Ref	28	6.86	1.07(0.54–2.11)	394	5.56	0.97(0.55–1.72)
Male	18	10.1	Ref	30	8.11	0.80(0.45–1.44)	411	6.88	0.76(0.47–1.21)

**Table 4:** Risk of herpes zoster complications (ocular) in different types of herpes zoster stratified by age and sex. Adjusted HR: Adjusted for age, sex, CAD, hypertension, hyperlipidemia, DM, stroke, AF, renal disease, heart failure, cancer, chronic hepatitis, and SLE. Rate: incidence rate per 1000 person-years.

Variable	HZO			HZN			HZT		
	Event	Rate	Adjusted HR (95% CI)	Event	Rate	Adjusted HR (95% CI)	Event	Rate	Adjusted HR (95% CI)
Age group									
<45	-	-	-	8	4.32	ref	31	0.79	0.20(0.09–0.43)
45–64	-	-	-	12	3.45	ref	73	1.25	0.37(0.20–0.69)
≧65	-	-	-	9	3.45	ref	101	2.79	0.86(0.44–1.71)
Sex									
Female	-	-	-	12	2.89	ref	102	1.41	0.54(0.30–0.99)
Male	-	-	-	17	4.49	ref	103	1.68	0.40(0.24–0.68)

**Table 5:** Risk of herpes zoster complications (neurological) in different types of herpes zoster stratified by age and sex. Adjusted HR: Adjusted for age, sex, CAD, hypertension, hyperlipidemia, DM, stroke, AF, renal disease, heart failure, cancer, chronic hepatitis, and SLE. Rate: incidence rate per 1000 person-years.

Variable	HZO			HZN			HZT		
	Event	Rate	Adjusted HR (95% CI)	Event	Rate	Adjusted HR (95% CI)	Event	Rate	Adjusted HR (95% CI)
Age group									
<45	9	8.60	ref	-	-	-	188	4.89	0.54(0.28–1.06)
45–64	30	23.6	ref	-	-	-	722	13.2	0.55(0.38–0.80)
≧65	43	33.8	ref	-	-	-	975	30.6	0.89(0.66–1.21)
Sex									
Female	32	16.3	ref	-	-	-	984	14.5	0.92(0.65–1.31)
Male	50	30.7	ref	-	-	-	901	15.7	0.62(0.46–0.82)

**Table 6:** Risk of herpes zoster complications (primary trigeminal neuralgia) in different types of herpes zoster stratified by age and sex. Adjusted HR: Adjusted for age, sex, CAD, hypertension, hyperlipidemia, DM, stroke, AF, renal disease, heart failure, cancer, chronic hepatitis, and SLE. Rate: incidence rate per 1000 person-years.

Variable	HZO			HZN			HZT		
	Event	Rate	Adjusted HR (95% CI)	Event	Rate	Adjusted HR (95% CI)	Event	Rate	Adjusted HR (95% CI)
Age group									
<45	5	4.63	ref	14	7.56	1.48(0.53–4.12)	177	4.55	0.89(0.37–2.18)
45–64	12	8.75	ref	38	11.1	1.22(0.64–2.33)	464	8.12	0.92(0.52–1.64)
≧65	22	15.7	ref	46	18.4	1.12(0.67–1.86)	522	15.0	0.95(0.62–1.46)
Sex									
Female	23	11.2	ref	59	14.6	1.23(0.76–1.99)	663	9.39	0.85(0.56–1.28)
Male	16	8.90	ref	39	10.4	1.18(0.66–2.11)	500	8.32	1.08(0.66–1.78)

**Table 7:** Risk of herpes zoster complications (other visceral) in different types of herpes zoster stratified by age and sex. Adjusted HR: Adjusted for age, sex, CAD, hypertension, hyperlipidemia, DM, stroke, AF, renal disease, heart failure, cancer, chronic hepatitis, and SLE. Rate: incidence rate per 1000 person-years.

Variable	HZO			HZN			HZT		
	Event	Rate	Adjusted HR (95% CI)	Event	Rate	Adjusted HR (95% CI)	Event	Rate	Adjusted HR (95% CI)
Age group									
<45	43	48.7	Ref	116	91.8	1.68(1.17–2.39)	-	-	-
45–64	78	78.5	Ref	273	144	1.55(1.21–2.00)	-	-	-
≧65	108	109	Ref	262	186	1.34(1.07–1.68)	-	-	-
Sex									
Female	108	67.9	Ref	350	149	1.76(1.41–2.18)	-	-	-
Male	121	94.9	Ref	301	135	1.24(1.00–1.53)	-	-	-

## Discussion

According to our review of relevant literature, this is the first population-based study clarifying the relationship between the initial presentation sites and HZ complications. We observed that the overall cumulative incidence of HZ complications in each HZ cohort was clinically significant (log-rank test, p < 0.0001), with the highest and lowest complication rates in the HZN and HZT cohorts, respectively (Fig 1 and Table 2). In this study, the HZN cohort was associated with a higher risk of ocular complications than was the HZT cohort (Table 2). Moreover, the HZO cohort showed a higher risk of neurological complications than did the HZT cohort (Table 2). Few large-scale studies have focused on the relationship between initial presentation sites and HZ complications. We conducted a literature review and found two similar population-based cohort studies in Taiwan that had documented an increased risk of stroke after HZO [10, 28]. One study reported that the adjusted HRs for stroke after HZ and HZO during the 1-year follow-up period were 1.31 and 4.28, respectively. The other study compared 658 patients with HZO and 1974 controls and reported a mean 4.52-fold increased risk of stroke [10, 28]. Because the neurological complications may occur several months after an HZO event, the association is often overlooked. In this study, we indicated the relevance of this association to facilitate early detection. Our findings highlight that the initial presentation site can be an early predictor of neurological and ocular complications of HZ. However, additional studies are warranted to confirm our findings and explore the underlying pathogenesis.

Furthermore, to eliminate possible confounders, the association of initial presentation sites with complications was adjusted by comorbidities. We also analyzed the effects of sex and age by using stratification analyses and observed an increased risk of these complications in older age groups, which is compatible with previous studies [5, 29]. Our results revealed no significant difference between men and women in the type of HZ complication (Tables 3–7). Like previous studies, [1, 11, 30], we could not ascertain the relationship between sex and the incidence of HZ complications. We reason that decreased cell-mediated immunity leads to VZV reactivation [14, 29]. Elderly patients with age-related decreased cell-mediated immunity or immunocompromised patients, including recipients of organ or hematopoietic stem-cell transplants; those receiving immunosuppressive therapy; and those with lymphoma, leukemia, or HIV infection, have an increased risk of HZ [1, 31, 32]. Cancer patients with relatively impaired immunity develop significantly more nonpainful HZ symptoms but an equal number of painrelated HZ complications [33]. Our results regarding the initial presentation sites revealed significant differences in nonpainful complications (ocular, neurological, and other visceral complications) but not in PHN and cutaneous complications. Although the mechanism of this phenomenon remains unknown, some studies have speculated on the basis of herpes virus that the mechanism involves retrograde axonal transport within the oculomotor nerve to the brain or along the optic nerve to the eyes [34–36]. Additional studies are warranted to elucidate the mechanism through which the locations of HZ reactivation are determined.

The use of the LHID strengthens the validity of this study in the following aspects. First, this study had a large sample size (24,543 patients with HZ) and a long-term follow-up period (10 years), which extensively increased its statistical power. Second, this study is highly representative of the Taiwanese population because the NHI program covers 99% of the 23 million residents and has contracts with 97% of hospitals and clinics in Taiwan [37, 38]. These hospitals and clinics are densely distributed, highly accessible, and charge very low fees. All beneficiaries are eligible for receiving medical services by paying a nominal copayment of US $3–$15 for each visit [39]; therefore, most patients with a painful disease such as HZ are strongly motivated to visit physicians.

Several limitations of this study should be addressed. First, potential miscoding and misdiagnosis may have hampered the present results. Nevertheless, all insurance claims in the NHI program are filed by board-certified physicians, scrutinized by medical reimbursement specialists, and subjected to peer review. In addition, considering the distinctive appearance of HZ, the diagnosis of HZ is relatively simple. Furthermore, we excluded some symptom-based diagnosis codes, such as ICD-9-CM 729.2 for neuralgia, 784.0 for facial pain, and 698.9 for itching. Therefore, the diagnoses and codes for HZ infection should be accurate and reliable. Data from the NHI program have been validated and applied widely in several HZ epidemiological studies [4, 30, 40, 41]. Second, in previous studies, approximately 2.3%–6.2% of patients experienced HZ recurrence during a follow-up period of 8–10 years [42, 43]. A review of 859 patients revealed that 83% of them had one complication, 12% had two complications, and 5% had three or more complications [44]. In this study, we could not determine whether the second HZ event was a recurrence or complication; therefore, complications at the initial presentation sites were excluded to prevent this type of misclassification. Third, although we adjusted for the comorbidities and basic demographic data of the patients in the analysis, other factors reportedly associated with HZ, such as psychological status, previous trauma, and prodromal pain, were not observed in this study [21, 45, 46]. The relationship among these factors was not recorded in the LHID; thus, these factors were not included in our study design.

In summary, our results revealed that the initial presentation sites might be highly associated with subsequent complication sites. Our results are expected to benefit physicians and increase their awareness regarding HZ complications and different presentation sites. This study is the first to investigate epidemiological data on the relationship between the initial presentation sites and complication sites of HZ. Additional studies are required to provide conclusive results regarding the predictors of HZ complications.
